# Comparison of early and late ^68^Ga-FAPI-46-PET in 33 patients with possible recurrence of pancreatic ductal adenocarcinomas

**DOI:** 10.1038/s41598-023-43049-2

**Published:** 2023-10-19

**Authors:** Jorge Hoppner, Levin van Genabith, Thomas Hielscher, Ulrike Heger, Lucas Sperling, Teresa Colbatzky, Ewgenija Gutjahr, Matthias Lang, Thomas Pausch, Anna-Maria Spektor, Frederik M. Glatting, Jakob Liermann, Thilo Hackert, Clemens Kratochwil, Frederik L. Giesel, Uwe Haberkorn, Manuel Röhrich

**Affiliations:** 1 Department of Nuclear Medicine, University Hospital Heidelberg, Im Neuenheimer Feld 400, 69120 Heidelberg, Germany; 2 Department of Biostatistics, German Cancer Research Center, Heidelberg, Germany; 3 Department of General, Visceral, and Transplantation Surgery, University Hospital Heidelberg, Heidelberg, Germany; 4 Department of Pathology, University Hospital Heidelberg, Heidelberg, Germany; 5 Clinical Cooperation Unit Molecular and Radiation Oncology, German Cancer Research Center (DKFZ), Heidelberg, Germany; 6 Department of Radiation Oncology, University Hospital Heidelberg, Heidelberg, Germany; 7 Department of Nuclear Medicine, University Hospital Düsseldorf, Düsseldorf, Germany; 8 Clinical Cooperation Unit Nuclear Medicine, German Cancer Research Center (DKFZ), Heidelberg, Germany; 9 Department of Nuclear Medicine, University Hospital Mainz, Langenbeckstraße 1, 55131 Mainz, Germany

**Keywords:** Gastrointestinal diseases, Molecular medicine

## Abstract

Positron emission tomography with ^68^Gallium (^68^Ga) labeled inhibitors of fibroblast activation protein (^68^Ga-FAPI-PET) is a promising imaging technique for patients with recurrent pancreatic ductal adenocarcinomas (PDAC). To date, it is not clear if different acquisition timepoints for ^68^Ga-FAPI-PET may result in comparable imaging information and if repetitive ^68^Ga-FAPI-PET imaging may add diagnostic value to single timepoint acquisition for recurrent PDAC. Here we analyzed retrospectively early (20 min p.i.) and late (60 min p.i.) ^68^Ga-FAPI-PET imaging using FAPI-46 of 33 patients with possible recurrence of PDAC concerning detection rates and uptake over time of local recurrences, metastases, inflammatory lesions of the pancreas, cholestatic lesions of the liver and reactive tissue. 33 patients with histologically confirmed PDAC after complete or partial resection of the pancreas and possible recurrence were examined by ^68^Ga-FAPI-46-PET acquired 20- and 60-min post injection (p.i.) of the radiotracer. FAPI-positive lesions were classified as local recurrences, metastases, inflammatory lesions of the pancreas (ILP), cholestatic lesions of the liver and reactive tissue based on histology, PET- and CT-morphology and clinical information. Lesions were contoured, and standardized uptake values (SUVmax and SUVmean) and target-to-background ratios (TBR) were analyzed for both acquisition timepoints. In total, 152 FAPI-positive lesions (22 local relapses, 47 metastases, 26 inflammatory lesions of the pancreas, 28 reactive tissues, and 29 cholestatic lesions) were detected. Detection rates for the early and late acquisition of ^68^Ga-FAPI-46-PET were almost identical except cholestatic lesions, which showed a higher detection rate at early imaging. SUV parameters and TBRs of ILP significantly decreased over time. Cholestatic lesions showed a tendency towards decreasing uptake. All other types of lesions showed relatively stable uptake over time. Early and late acquisition of ^68^Ga-FAPI-PET results in comparable imaging information in patients with possible recurrence of PDAC. Two timepoint imaging offers additional diagnostic potential concerning differential diagnoses.

## Introduction

Pancreatic ductal adenocarcinoma (PDAC) is an aggressive and highly lethal cancer with a 5-year survival rate of less than 10% despite surgical resection, radiotherapy, and chemotherapy^1^. A primary reason for the poor prognosis of PDACs is their high rate of local, regional or distant recurrence (80% after radical surgical resection)^2^. The early and accurate detection of recurrence, including adequate TNM staging, is crucial for optimal treatment and clinical management of patients with recurrent PDAC. For follow-up examinations of PDAC, contrast-enhanced computed tomography (ceCT) represents the clinical gold standard^1,3^ which is frequently complemented by transabdominal ultrasonography^4^ and magnetic resonance imaging MRI^5^ in clinical routine.

Recently, we could demonstrate that positron emission tomography with ^68^Gallium (^68^Ga) labeled fibroblast activation protein (FAP) inhibitors (^68^Ga-FAPI-PET) allows high contrast imaging of PDAC due to their prominent stroma containing FAP-positive cancer-associated fibroblasts. Furthermore, we showed that ^68^Ga-FAPI-PET imaging findings significantly alter TNM staging and oncological management of patients with PDAC compared to ceCT-based findings, particularly concerning recurrences^6^. In addition, a recent study demonstrated that ^68^Ga-FAPI-PET shows higher sensitivity in detecting primary pancreatic tumors, involved lymph nodes, and metastases and is superior in terms of TNM staging compared to ^18^Fluor-Desoxyglucose (^18^F-FDG)-PET/CT^7^. On the other hand, FAPIs are not exclusively tumor-specific tracers, and especially inflammatory lesions of remaining parts of the pancreas, reactive post-operative tissue, and hepatic cholestasis can be challenging for the interpretation of ^68^Ga-FAPI-PET images concerning suspected recurrence of PDAC^6,8,9^. In most studies on FAPI-PET, images were acquired one-hour post injection (p.i.) of the radiotracer (e.g. Refs.^10,11^). However, several recent studies suggest that earlier FAPI-PET acquisition timepoints lead to almost identical detection rates and similar target-to-background ratios (TBR) for various malignant tumors, including PDAC, compared to one-hour p.i.^8,12^. It has been demonstrated that malignant and non-malignant tissues may have a differential kinetic behavior in FAPI-PET, and it has been suggested that two- or multiple-timepoint imaging acquisition may facilitate their differentiation^6,12,13^. However, these studies applied variable protocols and included relatively small numbers of patients with different malignancies and clinical settings.

To evaluate dual time-point ^68^Ga-FAPI-PET imaging in a larger, more homogenous population of PDAC patients, we here retrospectively analyze early (20 min p.i.) and late (60 min p.i.) ^68^Ga-FAPI-PET using FAPI-46 of 33 patients with possible recurrence of PDAC. We compare imaging properties of malignant, inflammatory, reactive, and cholestatic lesions at two-timepoints in order to assess if early image acquisition is a possible alternative to acquisition one-hour p.i. Further, we evaluate the diagnostic potential of two-timepoint imaging for differentiating malignant from false-positive findings in the setting of possible recurrence of PDAC.

## Materials and methods

### Patient characteristics

Between 04/2021 and 02/2022, 33 patients (mean age 66.8 years, max 83, min 50; 17 male, 16 female) with possible recurrence after complete or partial resection of the pancreas and histological confirmation of PDAC were examined by ^68^Ga-FAPI-46-PET. All patients were referred based on an individual clinical indication to assist clinical decision-making by their treating physicians in the European Pancreas Center Heidelberg. Written informed consent was obtained from all patients on an individual basis, following the regulations of the German Pharmaceuticals Act §13(2b). Clinical characteristics and outcomes were collected through electronic patient records. The local institutional review board approved this retrospective analysis (study number S-115/2020). Table 1 gives a detailed patientwise overview of clinical characteristics of all patients.

**Table 1:** Patient characteristics of 33 patients with possible recurrence of PDAC.

Pat. number	Sex	Age	Histological diagnosis	Tumor localization	Resection status	Time between surgery and PET (days)	Systemic therapy
1	F	61	PDAC	Pancreatic tail	R0	207	none
2	F	72	PDAC	Pancreatic head	R0 (CRM +)	237	FOLFIRINOX
3	M	61	PDAC	Pancreatic head	R0 (CRM +)	368	FOLFIRINOX
4	M	75	PDAC	Pancreatic tail	R0	290	None
5	M	70	PDAC	Pancreatic head	R0	324	Gem/Cap
6	M	56	PDAC	Pancreatic head	R0	389	FOLFIRINOX
7	D	73	PDAC	Pancreatic tail	R0	320	None
8	D	61	PDAC	Pancreatic head	R0 (CRM +)	233	FOLFIRINOX
9	D	65	PDAC	Pancreatic head	R0 (CRM +)	216	FOLFIRINOX
10	M	62	PDAC	Pancreatic head	R0	366	FOLFIRINOX
11	D	62	PDAC	Total pancreas	R0 (CRM +)	280	FOLFIRINOX
12	M	52	PDAC	Pancreatic corpus	R1	407	FOLFIRINOX + Gem/Cap
13	M	66	PDAC	Pancreatic head	R1	507	FOLFIRINOX
14	D	80	PDAC	Pancreatic corpus	R0 (CRM +)	218	FOLFIRINOX
15	M	65	PDAC	Pancreatic head	R0	504	None
16	D	82	PDAC	Pancreatic corpus	R0	363	FOLFIRINOX + Gem mono
17	M	66	PDAC	Pancreatic head	R0	443	None
18	D	58	PDAC	Pancreatic head	R1	343	FOLFIRINOX
19	M	81	PDAC	Pancreatic tail	Rx	275	FOLFIRINOX
20	D	84	PDAC	Pancreatic head	R2	315	FOLFIRINOX
21	D	70	PDAC	Pancreatic head	R0	256	Gem mono
22	D	61	PDAC	Pancreatic head	R0	170	FOLFIRINOX
23	M	69	PDAC	Pancreatic head	R0 (CRM +)	162	FOLFIRINOX
24	M	50	PDAC	Pancreas, different parts	R1	535	None
25	F	83	PDAC	Pancreatic head	R1	293	None
26	M	66	PDAC	Pancreatic head	R0	337	Gem/Cap
27	M	73	PDAC	Pancreatic head	R0	505	FOLFIRINOX
28	M	58	PDAC	Pancreatic head	R0 (CRM +)	330	None
29	F	58	PDAC	Pancreatic head	R1	437	None
30	M	50	PDAC	Pancreatic corpus	R0	294	None
31	F	60	PDAC	Total pancreas	R0	320	None
32	F	75	PDAC	Pancreatic head	R1	588	Gem/Cap
33	M	74	PDAC	Pancreatic tail	R0	255	None

*f* female, *m* male, *PDAC* pancreatic ductal adenocarcinoma, *Gem* Gemcitabine, *Cap* Capecitabine, *FOLFIRINOX* Folin acid, 5-Fluoruracil, Irinotecan, Oxaliplatin.

### ^68^Ga-FAPI-46-PET/CT Imaging

Synthesis and labeling of ^68^Ga-FAPI-46 were conducted as previously described^14^. A Siemens Biograph mCT Flow scanner was used for PET imaging, according to previously published protocols^13^. In short, after a low-dose CT without contrast, 3-dimensional PET scans were acquired (matrix, 200 × 200), reconstructions were performed, and emission data was corrected for attenuation. For all patients, static PET scans were acquired 20- and 60-min post injection (p.i.) of 200–295 MBq of ^68^Ga-labeled FAPI-46.

### Image evaluation

For PET-Scans, SUVmax and SUVmean values, as well as target-to-background ratios (TBR) (versus mediastinal blood pool) 20 and 60 min p.i. of local recurrences of PDAC, metastatic lesions, inflammatory lesions of the pancreas, cholestatic lesions of the liver, reactive post-operative tissue and healthy organs, were analyzed. Visually discernible (20 and/or 60 min p.i.) ^68^Ga-FAPI-positive lesions were classified based on either histology, clinical information (clinical course during follow-up examinations, tumor marker Carbohydrate-Antigen 19–9 (CA 19–9) over time with respect to recurrence and inflammation markers such as C-reactive protein (CRP), lipase and amylase with respect to inflammatory lesions), CT morphology (space-occupying, progredient lesions with narrowing of surrounding vessels were considered typical for local recurrences, new, contrast enhancing lesions or progredient lymph nodes with pathological shape were considered typical metastatic, atrophy of the pancreas over time was considered as typical sign of chronic inflammation, diffuse- sector shaped hepatic contrast enhancement without focal mass lesion was considered typical for cholestatic lesions, masses in areas of surgery without growth, compression or infiltration of surrounding structures was considered typical for reactive tissue) or their appearance in ^68^Ga-FAPI-PET/CT (focal intense uptake was interpreted as typically malignant, diffuse or weak uptake was interpreted as typically reactive or inflammatory). Only lesions with CT-morphological correlates and highly suggestive characteristics for one of these classes were included. Possible FAPI-negative lesions were not considered for analysis. Pathological lesions and healthy organs were contoured using a volume of interest (VOI) technique. VOIs were defined by an automatic isocontour with a cutoff at 50% of SUVmax. For the calculation of detection rates, lesions with a TBR higher than 1.3 (calculated for SUVmax) were counted as positive. PET data analysis was done using PMOD software (PMOD Technologies Ltd.). JH, LVG, and MR performed the classification, contouring, and analysis of the lesions and organs in consensus.

### Statistical analysis

We performed descriptive analyses for patients and their tumor characteristics. Differences in FAPI-uptake (measured as SUVmax and SUVmean) of the lesions at both timepoints were determined using a linear mixed model with random patient effect to account for multiple lesions per patient using R package ime4^15^. P-values were adjusted for multiple testing using Holm’s correction. A p-value of less than 0.05 was defined as statistically significant.

### Ethics approval

All procedures performed in studies involving human participants followed the ethical standards of the institutional and/or national research committee and the 1964 Helsinki declaration and its later amendments or comparable ethical standards. The local institutional review board (Ethikkommission der medizinischen Fakultät Heidelberg) approved this retrospective study (study number S-115/2020).

### Consent to participate

Informed consent was obtained from all individual participants included in the study.

## Results

### Early and late ^68^Ga-FAPI-46 biodistribution in patients with recurrent PDAC

**Figure 1:**
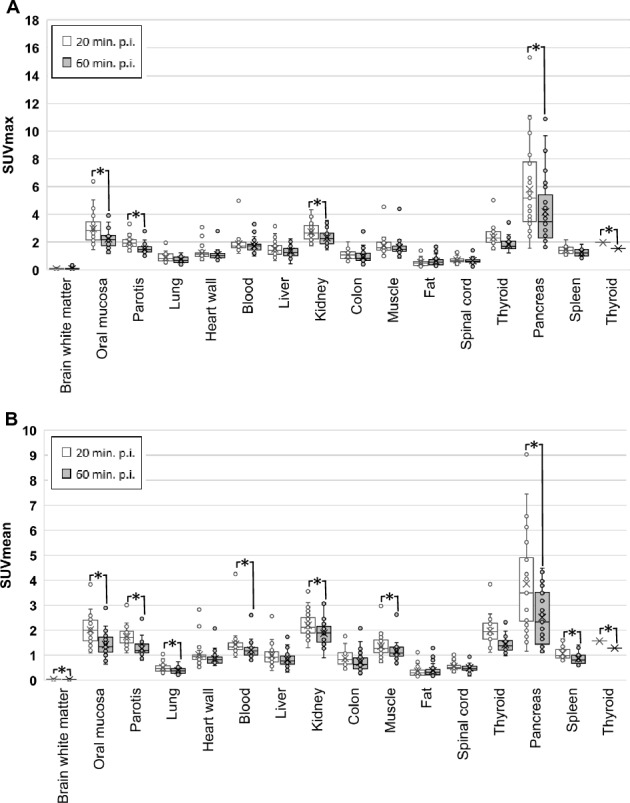
(**A**,**B**) Comparison of biodistribution (SUVmax (**A**) and SUVmean (**B**) + / − standard deviation) at early (20 min. p.i.) and late (60 min. p.i.) acquisition of static PET imaging with ^68^Ga-labeled FAPI-46 in 33 patients with possible recurrence of PDAC. Asterisks indicate significant (p-value < 0.05) differences between early and late uptake parameters.

All healthy organs showed low ^68^Ga-FAPI-46-uptake. The background activity of all tissues except fat tissue, which showed increasing uptake over time, was decreased when PET-Scans were performed 60 min p.i. compared to the earlier acquisition timepoint. Hereby, the differences between early and late uptake in terms of SUVmean were statistically significant in more organs than in terms of SUVmax. (Fig. 1A,B).

### Lesions and their classifications

In total, 152 FAPI-positive lesions in 31 patients were detected (two patients showed no FAPI-positive lesions and only background tissues were analyzed). 22 lesions were classified as local relapses, 47 as metastatic lesions (7 hepatic, 25 lymph nodes, one osseous, 14 peritoneal), 26 as inflammatory lesions of the pancreas (ILP), 28 as reactive tissue, and 29 as cholestatic lesions. Supplemental Table 1 gives a patientwise and lesionwise overview of all classified lesions and the respective basis of classification.

### Detection rates of early and late ^68^Ga-FAPI-46 acquisition

Cholestatic lesions showed a markedly higher detection rate at early imaging compared to late imaging (90% versus 66%). All other lesions showed almost similar detection rates at the two timepoints p.i:, local relapses 100% at both timepoints, metastatic lesions 100% at 20 min. p.i. and 94% at 60 min. p.i. (hepatic and osseous metastases 100% at both timepoints: peritoneal metastases 100% at 20 min p.i. and 93% at 60 min p.i.; lymph nodes metastases 100% at 20 min p.i. and 92% at 60 min. p.i.), inflammatory lesions of the pancreas 100% at both timepoints, reactive tissue: 79% at 20 min. p.i.; 93% at 60 min. p.i.. In Supplemental Table 1, the TBRmax value and at early and late acquisition is given for each lesion.

### Early and late ^68^Ga-FAPI-46 uptake of local recurrences, metastases, inflammatory lesions of the pancreas, cholestatic lesions, and reactive tissue

**Figure 2:**
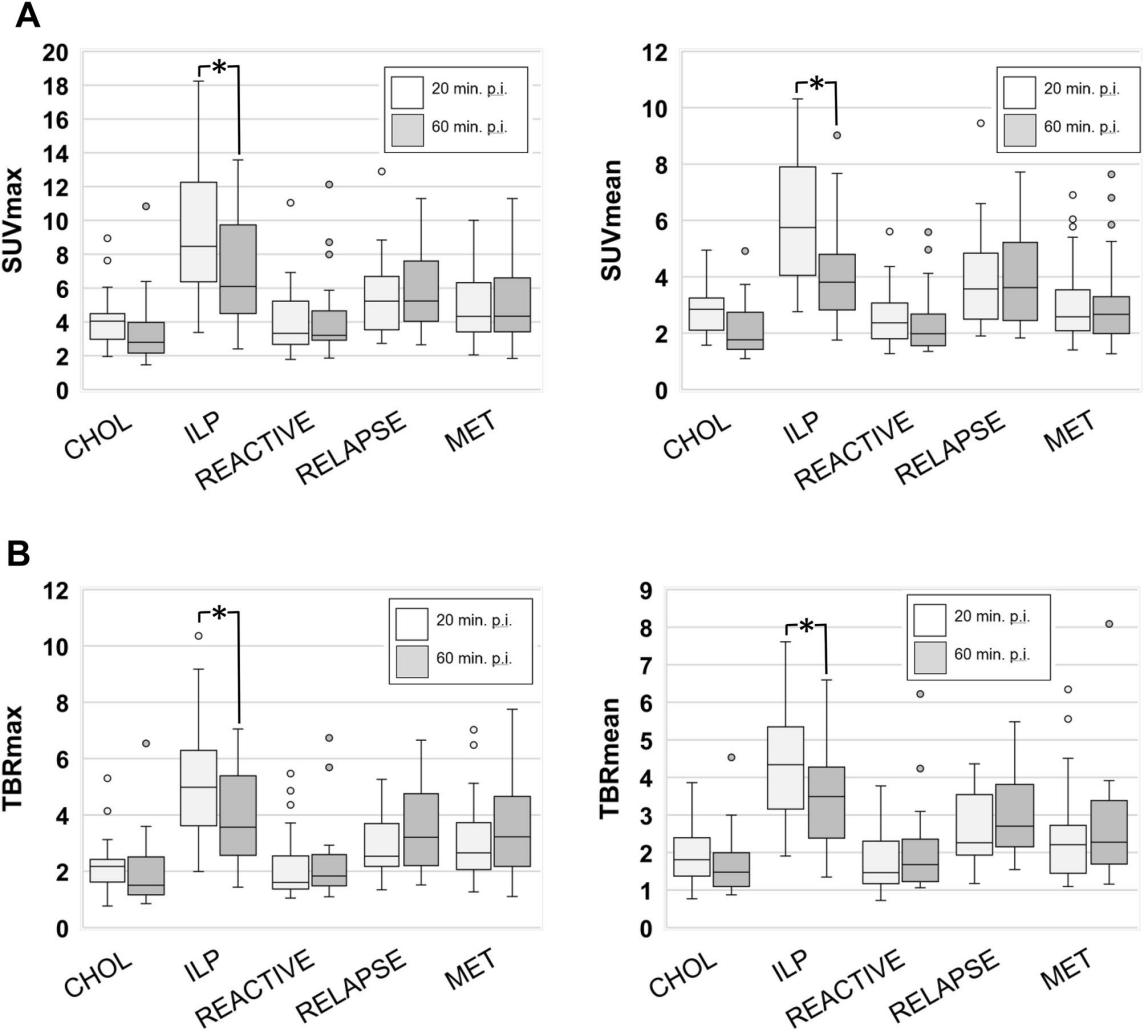
(**A**,**B**) Boxplots of SUVmax and SUVmean (**A**) values and corresponding TBR values (**B**) at early (20 min. p.i.) and late (60 min. p.i.) ^68^Ga-FAPI-46-PET acquisition of different types of lesions in 33 patients with possible recurrence of PDAC. *CHOL* cholestatic lesion, *ILP* inflammatory lesion of the pancreas, *MET* metastatic lesion). Boxes represent the interquartile range (IQR), whiskers the range of 1.5 IQR, and the horizontal line within the box indicates the median. Data outliers are shown separately within the graph. Asterisks indicate significant (p-value < 0.05) differences between early and late uptake parameters.

**Figure 3:**
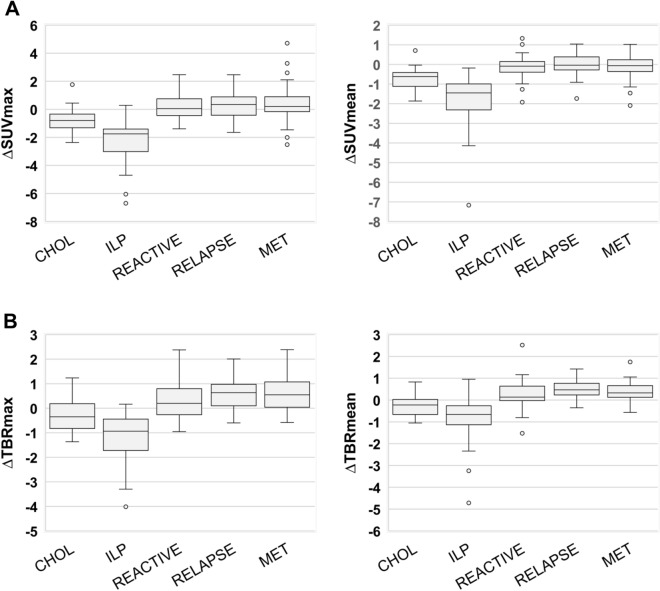
(**A**,**B**) Boxplots of the difference of SUVmax (ΔSUVmax) and SUVmean (ΔSUVmean) values (**A**) and corresponding tumor to background ratios (ΔTBR max) and ΔTBRmean) (**B**) of different types of lesions between early (20 min. p.i.) and late (60 min. p.i.) acquisition ^68^Ga-FAPI-46-PET in 33 patients with possible recurrence of PDAC. *CHOL* cholestatic lesion, *ILP* inflammatory lesion of the pancreas, *MET* metastatic lesion, *RT* reactive tissue, *REL* relapse). Boxes represent the interquartile range (IQR), whiskers the range of 1.5 IQR, and the horizontal line within the box indicates the median. Data outliers are shown separately within the graph.

**Figure 4:**
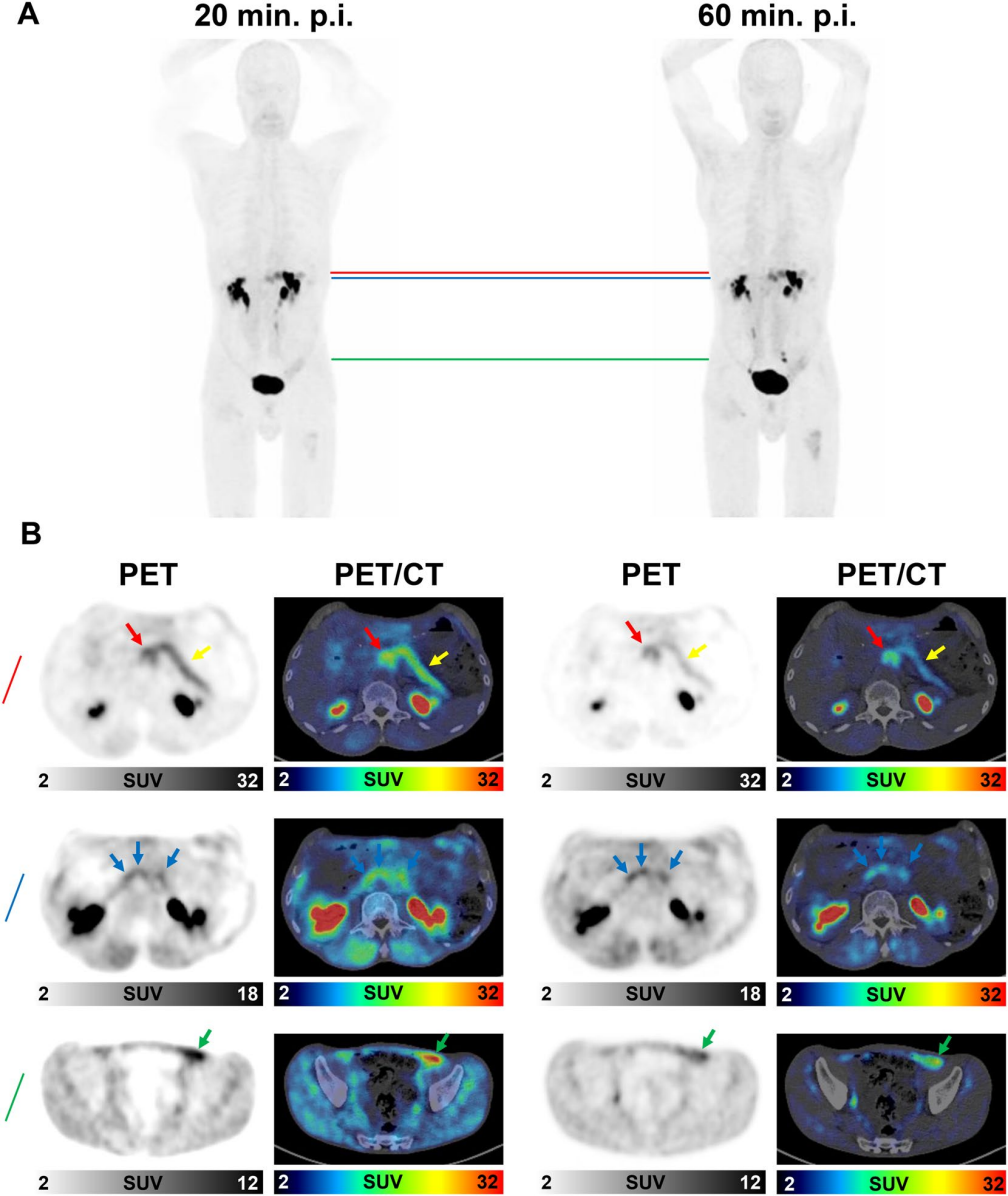
(**A**,**B**) ^68^Ga-FAPI-PET/CT imaging 20 and 60 min p.i. of a patient who had undergone resection of the pancreatic head and presented with local recurrence of a PDAC, an inflammatory lesion of the pancreas, suspicious paraaortic lymph nodes and suspected peritoneal carcinosis. (**A**) Maximum intensity projection images were acquired 20 and 60 min after the application of ^68^Ga-FAPI-46. The red line indicates the height of the local recurrence and the inflammatory lesion of the pancreas. The blue line indicates the height of three suspicious lymph nodes. The green line indicates the height of suspected peritoneal carcinosis. (**B**) Corresponding axial PET- and PET/CT images at the level of red, blue, and green line. The red arrow indicates the local recurrence. The yellow arrow indicates the inflammatory lesion of the pancreas. The blue arrows indicate the three suspicious paraaortic lymph nodes. The green arrow indicates the suspected peritoneal carcinosis. In the first row (red line), the differential uptake of the inflammatory lesion of the pancreas (decreasing over time) and the local recurrence (stable over time) lead to improved visual differentiation of both types of lesion. Suspicious lymph nodes and suspected peritoneal carcinosis are discernable in early and late imaging and show slightly improved target-to-background ratios in late imaging due to decreased background activity.

**Figure 5:**
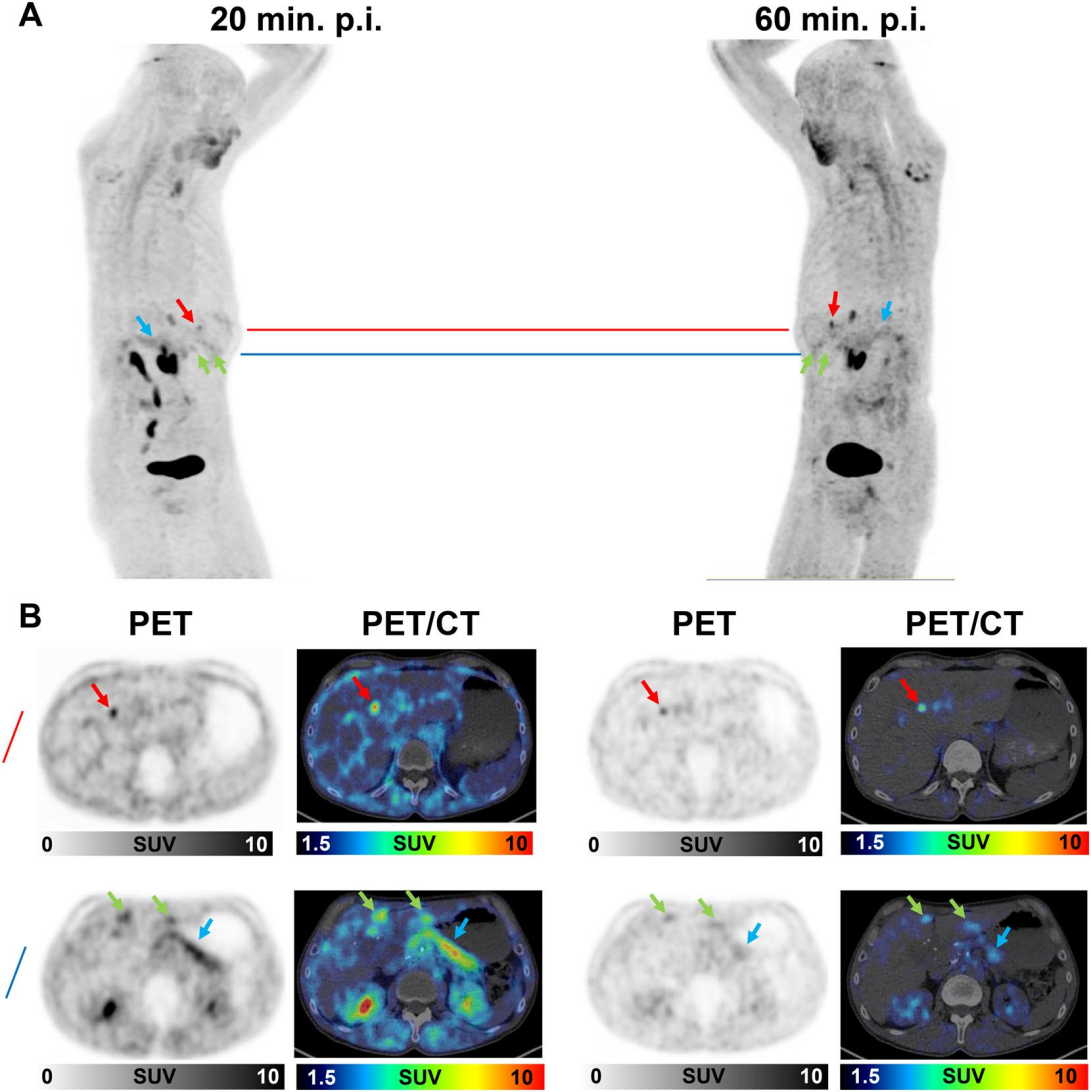
(**A**,**B**) ^68^Ga-FAPI-PET/CT imaging 20 and 60 min p.i. of a patient who had undergone whipple resection of the pancreatic head and presented with suspected liver metastases. (**A**) Maximum intensity projection images were acquired 20 and 60 min after the application of ^68^Ga-FAPI-46. The red line indicates a liver metastasis in liver segment IVa which was confirmed by morphological correlation in CT and MRI during follow up. The blue line indicates two cholestatic lesions in liver segment IVb and liver segment 3 without morphological correlates during follow-up and an inflammatory lesion of the remaining pancreatic tail. (**B**) Corresponding axial PET- and PET/CT images at the level of red and blue line. The red arrow indicates the liver metastasis. The green arrows indicate the cholestatic lesions of the liver and the blue arrow indicates the inflammatory lesion of the remaining pancreatic tail. The differential uptake of the liver metastasis (mostly stable over time and even better discernable due to reduced background activity) and the cholestatic lesions (decreasing over time, reduced discernibility in late imaging) can assist the interpretation of these FAPI-positive lesions. The inflammatory lesion of the pancreatic tail showed decreasing uptake over time, similar as in the patient presented in Fig. 4.

Comparing early and late ^68^Ga-FAPI-46-uptake of the different types of lesions, we found the highest early and late average SUVmax and SUVmean values in ILP, followed by local recurrences and metastatic lesions, whereas reactive tissues and cholestatic lesions of the liver showed relatively low uptake at both timepoints (Fig. 2A). SUV values and TBRs of ILP decreased significantly over time and TBRs of cholestatic lesions had tendency towards decreasing TBRs. TBRs of reactive tissues, local recurrences and metastases were relatively stable (Fig. 2B). Of note, uptake values over time of hepatic metastases, lymph node metastases, osseous metastases, and peritoneal metastases showed no significant differences relative to each other (Supplemental Fig. 1). Regarding the differences in uptake values between early and late ^68^Ga-FAPI-46-PET acquisition, ILP showed negative differential values (ΔSUVmax, ΔSUVmean, and ΔTBR). Cholestatic lesions showed a tendency towards negative differential values and all other types of lesions showed differential values of nearly zero (Fig. 3A,B). Figure 4 shows an exemplary case of a patient with a local recurrence of PDAC, ILP, suspected lymph node metastases, and suspected peritoneal carcinosis. Two-timepoint imaging showed differential early and late ^68^Ga-FAPI-46 uptake of these lesions, leading to improved differentiation between local recurrence and ILP. Figure 5 shows an exemplary case of a patient with liver metastasis and cholestatic lesions, in which the differential uptake over time facilitated the interpretation of FAPI-positive liver lesions.

## Discussion

### Summary of the results

We have retrospectively analyzed early (20 min p.i.) and late (60 min p.i.) ^68^Ga-FAPI-46-PET scans of 33 patients with possible recurrence of PDAC. We observed low background activities for both acquisition timepoints and almost identical detection rates for local and metastatic recurrences of PDAC and non-malignant lesions (inflammatory lesions of the pancreas, and reactive tissue) in early and late imaging—apart from the cholestatic lesions which were better detectable in early imaging. Inflammatory lesions of the pancreas showed a markedly reduced signal intensity and cholestatic lesions showed a slightly reduced signal intensity in late imaging compared to early imaging, while all other types of lesions showed mostly stable FAPI-46-uptake. According to this differential behavior over time, two-timepoint imaging improved the differentiation of ILP and local recurrences compared to PET acquisition at one of the timepoints. A potential explanation for our main finding, namely the decreasing signal intensity of inflammatory lesions in contrast to all other types of lesions could be the strong perfusion of inflammatory lesions that causes more rapid tracer influx as well as more rapid washout than in other types of lesions. Furthermore, differential FAPI-uptake kinetics of different subsets of activated fibroblasts in inflammatory and malignant lesions may contribute to the differential signal intensities in inflammatory and other lesions. However, further research on the underlying biological mechanisms of FAPI-avidity in different types of pathology is necessary to substantiate these hypotheses.

### FAPI-PET Imaging over time

Recently, our group and others have published studies on multiple timepoint FAPI-PET imaging. Ferdinandus et al. reported equivalent detection rates for primary and metastatic malignant lesions in 69 patients with different malignancies, 18 with pancreatic tumors examined by ^68^Ga-FAPI-46-PET and recommend early FAPI-PET acquisition (10 min p.i.) as a time- and cost-effective alternative to acquisition 60 min p.i.^8^. Conversely, Hu et al. analyzed early dynamic and repetitive static ^18^Fluor (^18^F)-FAPI-42-PET imaging of 22 patients and argued for acquisition 60 min p.i. due to optimal TBR values of malignant lesions^16^. In our recently published analysis of 24 patients examined by repetitive, 5-timepoint early FAPI-PET using FAPI-02, FAPI-46, and FAPI-74, we found equivalent detection rates of malignant, inflammatory/reactive and degenerative lesions by acquisition from 34 min p.i. up to 58 min p.i. and increasing TBR values over time for most types of lesions^12^. Our recent findings align with these analyses of repetitive FAPI imaging published so far and point out the additional diagnostic value of repetitive PET imaging in suspected recurrence of PDAC, where non-malignant FAPI-uptake can impair the interpretation of FAPI-PET images.

### Implications for follow-up examinations of PDAC

The high clinical potential of FAPI-PET imaging for PDAC, especially concerning tumor staging, has been demonstrated by a pilot study of our group. Notably, in the setting of recurrent PDAC, FAPI-PET appeared to impact staging and clinical decision-making significantly^17^. After these promising initial results, several prospective clinical trials are ongoing (NCT05262855, NCT05275985, NCT05518903) in order to generate clinical evidence for the application of ^68^Ga-FAPI tracers in PDAC. These studies are all focused on settings with primary PDAC, but based on our initial observations re-staging of PDAC with ^68^Ga-FAPI-PET would be a similarly attractive—if not even more promising—approach. owever, despite all enthusiasm, interpreting FAPI-positive lesions remains challenging as reactive, fibrotic, degenerative, and inflammatory lesions can be intensively FAPI-positive^6,13,18^. In our previous publications, we observed indications for differential tracer uptake over time of malignant and benign lesions as well for PDAC and ILP as for other organs like the lung in small numbers of patients who had undergone FAPI-PET at more than one timepoint^6,13^. Our results substantiate our initial observations of PDAC and ILP in a higher number of patients and strengthen the rationale for imaging at two or more timepoints in the recurrent PDAC. With respect to clinical workflow and time and cost-effective imaging, standard early imaging followed by late imaging in challenging cases (e.g. ILP versus local recurrence or cholestatic lesions versus liver metastases) would be a possible compromise. On the other hand, no clear diagnostic value of two-timepoint imaging compared to single timepoint imaging for the differentiation of other clinically relevant diagnostic problems like reactive tissue versus local recurrences or metastatic lesions could be demonstrated in this analysis as all of these lesions showed relatively stable uptake over time. Concerning these differential diagnoses, dynamic imaging displaying early differences (within the first 10–20 min) of ^68^Ga-FAPI-uptake may improve diagnostic accuracy and should be evaluated in future studies. In a recent project on dynamic and static ^68^Ga-FAPI-74 imaging of PDAC and intraductal papillary mucinous neoplasms (IPMN) of the pancreas, we observed delayed time to peak and differential k1 and k2 values after kinetic modeling for malignant PDAC and premalignant high-grade IPMN compared to low-grade IPMN, which are considered benign lesions^19^. According to these findings, we consider the application of dynamic FAPI-PET in suspected recurrences of PDAC a promising future approach for differentiating malignancy-associated and non-malignant FAPI-uptake. Next to modified PET acquisition protocols, the integrated analysis of PET data and innovative MRI parameters like diffusion weighted imaging (DWI), which can display functional and microarchitectural aspects like fibrosis, dell density or edema^20,21^ is a promising approach for future studies on ^68^Ga-FAPI-PET in PDAC.

### Dual time point imaging with other PET tracers

The concept of dual time point imaging has also been applied to other tracers than FAPIs: Numerous studies have evaluated the potential diagnostic value of dual time point imaging with ^18^F-FDG-PET/CT in pancreatic cancers^22^ and various other malignancies such as lung cancers^23^ or breast cancers^24^, but dual time point ^18^F-FDG-PET has not entered clinical routine. Hereby, it is noteworthy that dual time point imaging for ^18^F-FDG is typically performed 60 min p.i. and as delayed imaging 120 min p.i or even later resulting in prolonged PET-protocols that impede its regular application. Similarly, dual time point imaging with ^68^Ga-PSMA tracers^25,26^ and ^68^Ga-DOTATOC tracers^27^ has been performed as delayed imaging in addition to standard acquisition times. Late additional imaging with these three tracers is based on increasing accumulation in tumors over time. In contrast, the FAPI signal of malignancies appears to be stable to slightly decreasing over time^12^. Given the rapid washout of FAPI tracers in normal tissues, early dual time point imaging protocols, which can be better integrated into clinical routine are feasible.

### Limitations

The major limitation of our analysis is that only one out of 33 patients included has undergone biopsy or surgery for definitive confirmation of our classification of ^68^Ga-FAPI-46-positive lesions. Although we considered the appearance of these lesions in CT and—if available in MRI and ultrasound—and the clinical course of the patients as valid classifications, a rest of uncertainty remains due to missing histology in the other cases. Beyond that, possible FAPI-negative lesions were not included into our analysis so that a calculation of the diagnostic accuracy of ^68^Ga-FAPI-PET for the differentiation of different types of lesions is not possible based on this dataset. Another limitation arises from the retrospective nature of this project, which means that the patients analyzed were referred to ^68^Ga-FAPI-46-PET by their treating physicians on an individual basis and not according to a rigid study protocol, which may cause a certain heterogeneity in the population regarding their clinical characterization. Taken together, the findings of this retrospective analysis should be interpreted with caution and need definitive confirmation in systematic prospective studies.

## Conclusion

Early (20 min p.i.) and late (60 min p.i.) acquisition of ^68^Ga-FAPI-46-PET for patients with possible recurrence of pancreatic ductal adenocarcinomas resulted in almost identical detection rates for malignant and non-malignant lesions indicating that early acquisition of ^68^Ga-FAPI-46-PET is feasible. However, two-timepoint imaging offers additional diagnostic value concerning differential diagnoses, especially for the differentiation of ILP and local recurrences of PDAC. For patient as well as time and cost management we recommend early image acquisition (20 min p.i.) and facultative late image acquisition (60 min p.i.) for further differentiation of unclear FAPI-positive findings.

### Supplementary Information

Supplementary Information.

## Data Availability

The datasets generated during and/or analysed during the current study are available from the corresponding author on reasonable request*.*
