# Supplementary material for: Comparison of early and late 68Ga-FAPI-46-PET in 33 patients with possible recurrence of pancreatic ductal adenocarcinomas
Source: Sci Rep. 2023 Oct 19;13:17848. doi: 10.1038/s41598-023-43049-2 (PMC10587145; doi:10.1038/s41598-023-43049-2)
Supplement: Supplementary file 1 — Supplementary Information. [file 41598_2023_43049_MOESM1_ESM.docx]

**Comparison of early and late ^68^Ga-FAPI-46-PET in 33 patients with possible recurrence of pancreatic ductal adenocarcinomas**

**Running title: Early and late ^68^Ga-FAPI-PET for possible recurrence of PDAC**

Jorge Hoppner^1^, Levin van Genabith^1^, Thomas Hielscher^2^, Ulrike Heger^3^, Lucas Sperling^3^, Teresa Colbatzky^3^, Ewgenija Gutjahr^4^, Matthias Lang^3^, Thomas Pausch^3^, Anna-Maria Spektor^1^, Frederik M. Glatting^1^, Jakob Liermann^6^, Thilo Hackert^3^, Clemens Kratochwil^1^, Frederik L. Giesel^7^, Uwe Haberkorn^1^ and Manuel Röhrich^1^

**SUPPLEMENTAL MATERIAL**

**Supplemental Table 1: Lesionwise classification and detectability in ^68^Ga-FAPI-46-PET 20 and 60 minutes p.i. of 152 lesions of 31 patients with suspected recurrences of PDAC**

| Lesion number | Patient number | Localization | Classification | Basis of classification | TBRmax 20 min. p.i. | TBRmax 60 min. p.i. |
| --- | --- | --- | --- | --- | --- | --- |
| 1 | 1 | paraaortic right | local recurrence | clinical and radiological, | 2,589464 | 3,427415 |
| 2 | 1 | coeliac trunk | local recurrence | clinical and radiological | 1,348584 | 1,927194 |
| 3 | 1 | paraaortic left | local recurrence | clinical and radiological | 1,342007 | 1,850798 |
| 4 | 1 | liver segment 5 | CHOL | radiological | **0,827221** | **1,00574** |
| 5 | 1 | liver segment 8 | CHOL | radiological | **0,770759** | **1,147483** |
| 6 | 1 | periumbilical | reactive | radiological | **1,292089** | 2,703062 |
| 7 | 1 | left upper abdomen | MET_PER | clinical and radiological | **1,269838** | 1,31471 |
| 8 | 1 | left upper abdomen | MET_PER | clinical and radiological | **1,288155** | **1,182578** |
| 9 | 1 | intestinal | reactive | radiological | **1,088912** | 1,781759 |
| 10 | 1 | portal vein | MET_LN | clinical and radiological | 1,486115 | 2,286584 |
| 11 | 1 | portal vein | MET_LN | clinical and radiological | 1,383779 | 1,482473 |
| 12 | 1 | retrocrural right | MET_LN | clinical and radiological | 1,394458 | 2,104836 |
| 13 | 2 | pancreas | ILP | radiological | 3,999305 | 3,381985 |
| 14 | 2 | periumbilical | reactive | radiological | 1,48731 | 2,596738 |
| 15 | 2 | coeliac trunk | local recurrence | radiological | 2,479448 | 3,166498 |
| 16 | 2 | paracaval right | local recurrence | radiological | 2,65885 | 2,542985 |
| 17 | 2 | liver capsule | reactive | radiological | 1,944191 | 2,029315 |
| 18 | 2 | liver capsule | reactive | radiological | 1,893499 | 1,784579 |
| 19 | 3 | liver segment 7 | CHOL | radiological | 4,332606 | 3,32711 |
| 20 | 3 | liver | CHOL | radiological | 2,363669 | 1,413567 |
| 21 | 3 | liver segment 5 | CHOL | radiological | 2,358042 | 1,616202 |
| 22 | 3 | pancreas | ILP | radiological | 10,35848 | 6,345622 |
| 23 | 4 | portal vein | reactive | radiological | 1,407711 | 1,341406 |
| 24 | 4 | coeliac trunk | reactive | radiological | 1,622461 | **1,223288** |
| 25 | 5 | pancreas | ILP | radiological | 3,951706 | 3,188278 |
| 26 | 5 | liver | CHOL | radiological | 2,173958 | 1,713268 |
| 27 | 5 | laparotomy scar tissue | reactive | radiological | **1,193513** | 1,325067 |
| 28 | 6 | pancreas | ILP | radiological | 3,417334 | 2,425113 |
| 29 | 6 | pancreas | local recurrence | histological | 3,916707 | 4,3969 |
| 30 | 6 | perihepatic | MET_PER | histological | 7,025048 | 7,751455 |
| 31 | 6 | lower abdomen middle | MET_PER | histological | 6,483975 | 6,647689 |
| 32 | 6 | portal vein | reactive | radiological | 4,858169 | 5,690037 |
| 33 | 6 | paraaortic left | MET_LN | histological | 4,24564 | 5,0741 |
| 34 | 6 | portal vein | MET_LN | histological | 3,730766 | 6,118934 |
| 35 | 6 | mesenteric root | MET_LN | histological | 2,857185 | 4,398461 |
| 36 | 6 | precaval | MET_LN | histological | 2,783023 | 4,047116 |
| 37 | 7 | left lower lobe, lung | reactive | radiological | 2,704387 | 1,750413 |
| 38 | 7 | pancreas | ILP | radiological | 6,946025 | 5,63369 |
| 39 | 7 | liver segment 4a | CHOL | radiological | 1,906463 | 1,356515 |
| 40 | 7 | liver segment 4b | CHOL | radiological | 1,830686 | **0,851611** |
| 41 | 7 | liver segment 2 | CHOL | radiological | 1,654183 | **1,069439** |
| 42 | 8 | pancreas | ILP | radiological | 8,513267 | 5,216282 |
| 43 | 8 | pancreas | local recurrence | clinical and radiological | 2,403886 | 2,388231 |
| 44 | 8 | liver segment 4 | CHOL | radiological | 4,141456 | 2,774588 |
| 45 | 8 | liver segment 8 | CHOL | radiological | 2,868021 | 1,853139 |
| 46 | 8 | liver segment 6 | CHOL | radiological | 2,404433 | 1,498632 |
| 47 | 8 | retrocaval | MET_LN | clinical and radiological | 2,192769 | 1,818031 |
| 48 | 8 | paraaortic left | MET_LN | clinical and radiological | 1,400874 | **1,22085** |
| 49 | 8 | mesenteric root | MET_LN | clinical and radiological | 1,475673 | **1,105095** |
| 50 | 9 | pancreas | ILP | radiological | 6,09804 | 6,263489 |
| 51 | 9 | pancreas | ILP | radiological | 7,472187 | 7,052775 |
| 52 | 9 | liver segment 5 | MET_LIVER | radiological | 2,161381 | 2,175291 |
| 53 | 9 | liver segment 8 | CHOL | radiological | 2,188013 | 2,023444 |
| 54 | 9 | upper abdomen | MET_PER | radiological | 1,464572 | 1,821378 |
| 55 | 9 | coeliac trunk | local recurrence | radiological | 1,868674 | 2,105917 |
| 56 | 10 | pancreas | ILP | radiological | 2,746374 | 2,610944 |
| 57 | 10 | liver segment 4a | CHOL | radiological | 2,296947 | 2,486872 |
| 58 | 10 | resection area | local recurrence | radiological | 2,992017 | 3,686373 |
| 59 | 10 | paraaortic right | MET_LN | radiological | 2,206783 | 3,171369 |
| 60 | 10 | paraaortic left | MET_LN | radiological | 2,331208 | 4,656114 |
| 61 | 10 | mesenteric root | MET_LN | radiological | 1,667448 | 2,715035 |
| 62 | 11 | liver segment 4b | MET_LIVER | clinical and radiological | 2,184786 | 2,22412 |
| 63 | 11 | liver segment 3 | reactive | radiological | 1,439329 | 1,385172 |
| 64 | 11 | pancreas | ILP | radiological | 2,598461 | 2,155271 |
| 65 | 11 | port-a-cath. | reactive | radiological | 2,078019 | 1,884449 |
| 66 | 11 | lung | reactive | radiological | 1,73937 | **1,096406** |
| 67 | 12 | pancreas | ILP | radiological | 1,993634 | 1,559504 |
| 68 | 12 | resection area | reactive | radiological | 3,462951 | 2,899769 |
| 69 | 12 | liver segment 5 | CHOL | radiological | 1,41855 | **1,041337** |
| 70 | 12 | liver segment 4a | CHOL | radiological | **1,15086** | **1,019557** |
| 71 | 12 | liver segment 3 | reactive | histological | **1,05141** | 1,317807 |
| 72 | 12 | coeliac trunk | reactive | radiological | 1,556644 | 1,689763 |
| 73 | 13 | liver segment 7 | MET_LIVER | radiological | 2,46313 | 2,256226 |
| 74 | 13 | liver segment 4a | MET_LIVER | radiological | 2,259323 | 2,280874 |
| 75 | 13 | pancreas | ILP | radiological | 2,138567 | 1,436482 |
| 76 | 13 | liver segment 4b | CHOL | radiological | 1,74643 | 1,47092 |
| 77 | 14 | mesenteric root | MET_LN | radiological | 2,653356 | 3,290479 |
| 78 | 14 | anterior abdomen | MET_PER | radiological | 4,618453 | 6,568472 |
| 79 | 14 | anterior abdomen | MET_PER | radiological | 3,334555 | 3,326633 |
| 80 | 14 | resection area | MET_LN | radiological | 3,090662 | 3,86325 |
| 81 | 14 | upper abdomen left | local recurrence | radiological | 2,835658 | 3,620659 |
| 82 | 14 | renal artery left | MET_LN | radiological | 4,227819 | 5,826356 |
| 83 | 14 | aortic bifurcation | MET_PER | radiological | 2,902364 | 3,223147 |
| 84 | 14 | mesenteric root | MET_LN | radiological | 2,755131 | 3,267141 |
| 85 | 14 | mesenteric root | MET_LN | radiological | 2,508969 | 3,056834 |
| 86 | 14 | mesenteric root | MET_LN | radiological | 2,695729 | 3,55245 |
| 87 | 14 | mesenteric root | MET_LN | radiological | 2,570431 | 2,963513 |
| 88 | 14 | pancreas | local recurrence | radiological | 2,470096 | 3,249464 |
| 89 | 15 | resection area | local recurrence | radiological | 4,018956 | 5,989602 |
| 90 | 15 | pancreas | ILP | radiological | 5,734428 | 5,308041 |
| 91 | 15 | liver segment 7 | CHOL | radiological | 2,100462 | 2,18963 |
| 92 | 15 | liver | CHOL | radiological | 1,955449 | 2,53727 |
| 93 | 15 | left lower abdomen | MET_PER | radiological | 2,063051 | 2,796896 |
| 94 | 15 | left upper abdomen | MET_PER | radiological | 1,902671 | 2,195587 |
| 95 | 17 | resection area | reactive | radiological | 2,875675 | 2,585711 |
| 96 | 18 | pancreas | ILP | radiological | 6,675982 | 4,257565 |
| 97 | 18 | laparotomy scar | reactive | radiological | 5,467307 | 6,939111 |
| 98 | 18 | liver segment 5 | CHOL | radiological | 1,999968 | **1,269114** |
| 99 | 18 | liver segment 4 | CHOL | radiological | 2,207766 | **1,199086** |
| 100 | 18 | liver segment 2 | reactive | radiological | 1,524737 | 2,089446 |
| 101 | 19 | resection area | local recurrence | clinical and radiological | 5,264934 | 4,667253 |
| 102 | 19 | mesenteric root right | local recurrence | clinical and radiological | 3,434838 | 5,237675 |
| 103 | 19 | mesenteric root left | local recurrence | clinical and radiological | 2,200474 | 2,974262 |
| 104 | 20 | pancreas | ILP | radiological | 5,0216 | 3,223785 |
| 105 | 20 | pancreas | ILP | radiological | 4,528861 | 3,245504 |
| 106 | 20 | resection area | local recurrence | radiological | 3,623763 | 5,010876 |
| 107 | 20 | resection area | local recurrence | radiological | 1,581223 | 1,517895 |
| 108 | 20 | portal vein | CHOL | radiological | 1,586388 | **1,183194** |
| 109 | 20 | liver capsule | reactive | radiological | 1,358593 | 1,673909 |
| 110 | 21 | resection area | local recurrence | radiological | 2,20573 | 1,859727 |
| 111 | 21 | pancreas | ILP | radiological | 3,663371 | 2,181339 |
| 112 | 21 | pancreas | ILP | radiological | 3,471174 | 2,32317 |
| 113 | 21 | portal vein | CHOL | radiological | **1,26974** | **1,018007** |
| 114 | 21 | liver capsule | reactive | radiological | **1,233652** | 1,603561 |
| 115 | 22 | pancreas | ILP | radiological | 5,199912 | 4,847678 |
| 116 | 22 | coeliac trunk | reactive | radiological | 1,938903 | 2,450259 |
| 117 | 22 | mesenteric root | reactive | radiological | 1,473905 | 1,73926 |
| 118 | 22 | liver segment 5 | reactive | radiological | 4,358555 | 6,736081 |
| 119 | 23 | pancreas | ILP | radiological | 3,799319 | 2,915542 |
| 120 | 23 | liver segment 5 | CHOL | radiological | 5,303176 | 6,537949 |
| 121 | 23 | paraaortic left | MET_LN | radiological | 2,485487 | 2,061277 |
| 122 | 23 | left middle abdomen | MET_PER | radiological | 5,126062 | 6,787911 |
| 123 | 23 | left lower abdomen | MET_PER | radiological | 1,450828 | 1,920151 |
| 124 | 23 | resection area | local recurrence | radiological | 2,095014 | 2,231233 |
| 125 | 23 | resection area | local recurrence | radiological | 2,218594 | 2,49574 |
| 126 | 25 | pancreas | ILP | radiological | 6,162365 | 4,46447 |
| 127 | 25 | liver segment 8 | MET_LIVER | radiological | 3,266516 | 2,68552 |
| 128 | 25 | liver segment 5 | MET_LIVER | radiological | 3,689437 | 3,805109 |
| 129 | 25 | liver segment 3 | MET_LIVER | radiological | 2,404816 | 1,936761 |
| 130 | 26 | pancreas | ILP | radiological | 4,950766 | 3,159073 |
| 131 | 26 | liver segment 4 | CHOL | radiological | 2,657771 | 3,5921 |
| 132 | 27 | pancreas | ILP | radiological | 9,176628 | 6,544436 |
| 133 | 28 | pancreas | ILP | radiological | 5,339105 | 4,06646 |
| 134 | 29 | liver | CHOL | radiological | 3,125178 | 3,375196 |
| 135 | 29 | liver segment 2 | CHOL | radiological | 2,436932 | 2,087741 |
| 136 | 29 | pancreas | ILP | radiological | 4,305448 | 3,748218 |
| 137 | 29 | resection area | local recurrence | clinical and radiological | 4,647872 | 6,654398 |
| 138 | 29 | resection area | local recurrence | clinical and radiological | 4,566005 | 6,56212 |
| 139 | 29 | paraaortic | MET_LN | clinical and radiological | 4,96879 | 6,823975 |
| 140 | 29 | paraaortic | MET_LN | clinical and radiological | 4,513513 | 6,366434 |
| 141 | 29 | paraaortic | MET_LN | clinical and radiological | 4,514689 | 4,803652 |
| 142 | 29 | os sacrum right | MET_OSS | clinical and radiological | 2,957408 | 4,280233 |
| 143 | 29 | liver segment 7 | CHOL | radiological | 2,365563 | 2,67966 |
| 144 | 29 | mesenteric | MET_LN | clinical and radiological | 3,269871 | 4,342615 |
| 145 | 30 | resection area | reactive | radiological | 1,586914 | 2,473213 |
| 146 | 30 | liver | CHOL | radiological | 1,334006 | 1,50438 |
| 147 | 30 | right ventral abdomen | reactive | radiological | **1,156878** | 2,4509 |
| 148 | 31 | resection area | reactive | radiological | 1,813889 | 1,442494 |
| 149 | 32 | pancreas | HELP | clinical and radiological | 6,159812 | 5,691527 |
| 150 | 32 | umbilical | MET_PER | clinical and radiological | 3,905772 | 4,561695 |
| 151 | 32 | right ventral abdomen | MET_PER | clinical and radiological | 4,154161 | 6,397346 |
| 152 | 33 | resection area | reactive | clinical and radiological | 3,714379 | 2,926688 |
| Abbreviations: CHOL: inflammatory cholestatic lesion of the liver, MET_PER: metastatic lesion of the peritoneum, MET_LN lymph node metastasis, MET_LIVER: liver metastasis, ILP: inflammatory lesion of the pancreas, TBRmax: target to background ratio based on SUVmax values of lesions and aortic blood. Fat TBRmax values indicate lesions which were counted as not detectable for the calculation of detection rates. | | | | | | |

**Supplemental figure 1**

**
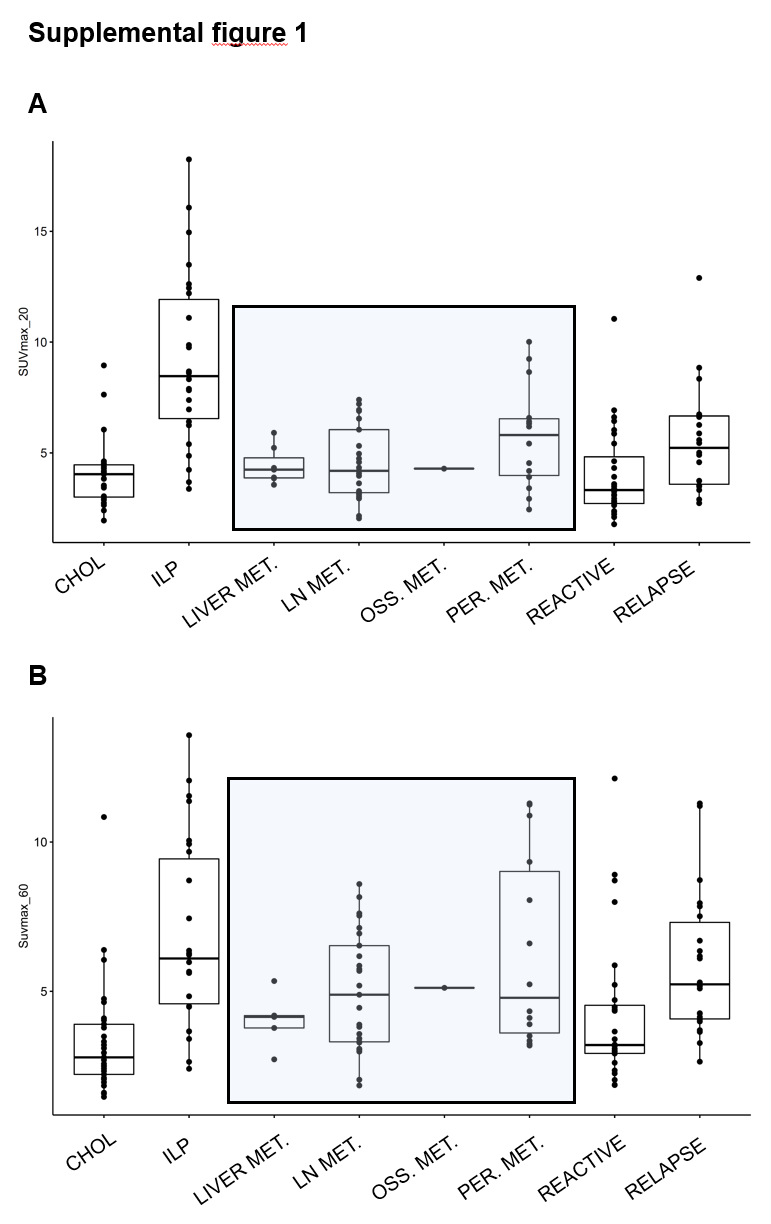
**

**
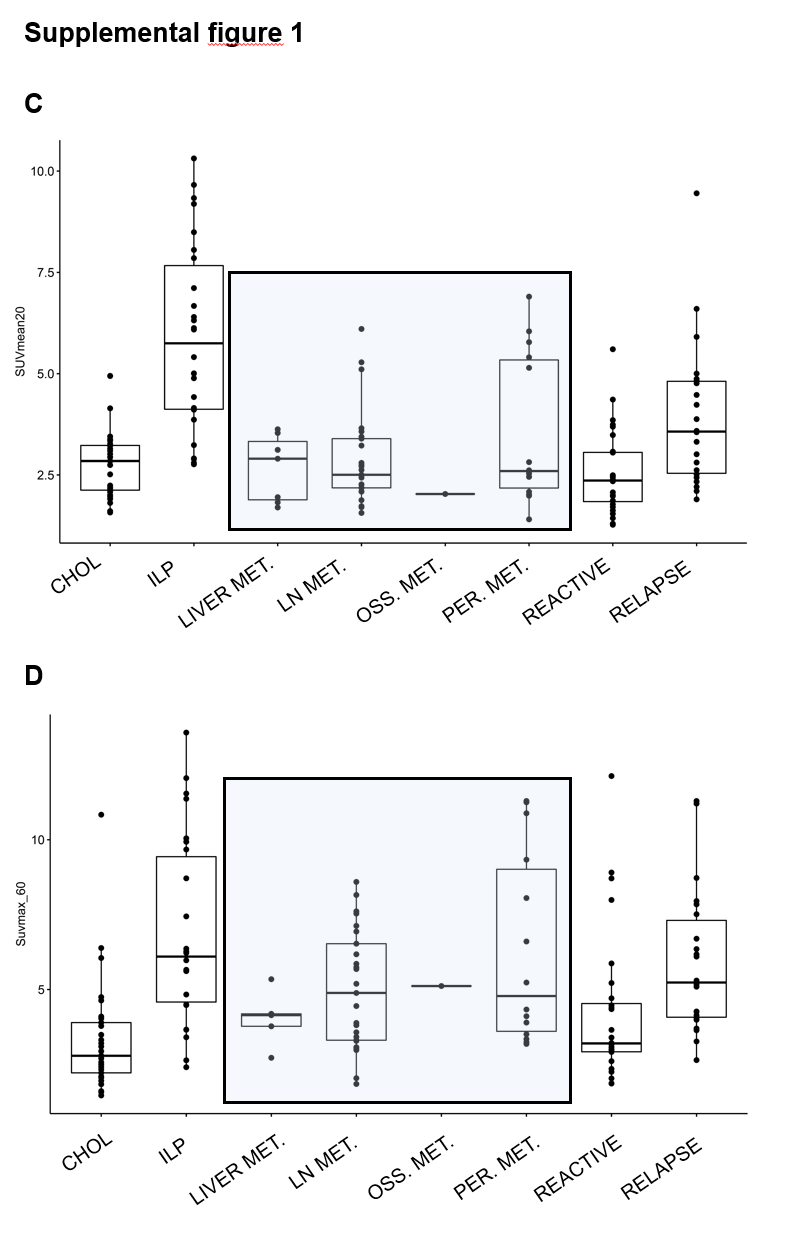
**

**A, B** Boxplots of early (A) and late (B) SUVmax and early (C) and late (D) SUVmean values derived from ^68^Ga-FAPI-46-PET of different types of lesions with subclassification of metastases due to locations (highlighted by boxes) in 33 patients with suspected recurrence of PDAC. Abbreviations: CHOL = cholestatic lesion, ILP= inflammatory lesion of the pancreas, LIVER MET. = metastatic lesion of the liver, LN MET. = Lymph node metastasis, OSS. MET. = osseous metastasis, PER. MET. = peritoneal metastasis). Boxes represent the interquartile range (IQR), whiskers the range of 1.5 IQR, and the horizontal line within the box indicates the median. Data outliers are shown separately within the graph.
